# Prevalence of Non-ST-Segment Elevation Myocardial Infarction in Acute Coronary Syndrome With Unremarkable Electrocardiogram: A Cross-Sectional Study From Lady Reading Hospital, Medical Teaching Institute, Peshawar

**DOI:** 10.7759/cureus.70575

**Published:** 2024-09-30

**Authors:** Usman Zeb, Nisar Ahmad Khan, Najeeb Ullah, Muhammad Hamza Khan

**Affiliations:** 1 Internal Medicine, Lady Reading Hospital, Medical Teaching Institute, Peshawar, PAK; 2 General Surgery, Lady Reading Hospital, Medical Teaching Institute, Peshawar, PAK

**Keywords:** acute coronary syndrome, cardiac emergencies, electrocardiogram (ecg/ekg), electrocardiography (ecg), inconclusive electrocardiogram, non st-elevation acute coronary syndrome, non-st segment elevation myocardial infarction, observational cross-sectional study, prevalance studies, unremarkable electrocardiogram

## Abstract

Introduction

Patients admitted to the cardiac care unit with non-ST-segment elevation myocardial infarction frequently have inconclusive electrocardiogram results. In individuals presenting with sudden central chest discomfort, non-ST-segment elevation myocardial infarction should be suspected until confirmed or ruled out by a cardiac biomarker analysis. This study aims to determine the prevalence of non-ST-elevation myocardial infarction among acute coronary syndrome patients who present with normal electrocardiogram findings.

Materials and methods

This study was conducted from October 24, 2022, to April 24, 2023, and involved 120 patients who came to the cardiac emergency department at Lady Reading Hospital, Peshawar. These patients experienced chest pain and symptoms of acute coronary syndrome, all within 24 hours of the onset of their symptoms. Demographic data and clinical histories were recorded for each patient. Electrocardiograms were performed, and non-ST-segment elevation was observed. The diagnosis of non-ST-segment elevation myocardial infarction was confirmed by measuring cardiac troponin T levels. A threshold value greater than 0.5 ng/mL was considered diagnostic. Data analysis was performed using the Statistical Package for the Social Sciences (SPSS) (IBM SPSS Statistics for Windows, Version 23, Armonk, NY), and grammar was checked using the Grammarly software (Grammarly, Inc., San Francisco, CA).

Results

The study participants had an average age of 53.96 years, with a standard deviation of 8.57. Most participants were male, comprising 65.83% of the total population, while 34.17% were female. Among the participants, 26.66% were diagnosed with non-ST-segment elevation myocardial infarction, affecting 32 individuals. The results showed a significant correlation between the occurrence of non-ST-segment elevation myocardial infarction and male gender, as well as with individuals who had pre-existing comorbidities. The p-values for these associations were 0.032 and 0.043, respectively, indicating statistically significant relationships.

Conclusion

In conclusion, a significant proportion of patients who presented to the emergency department with acute coronary syndrome symptoms and a normal electrocardiogram were diagnosed with non-ST-segment elevation myocardial infarction. This highlights the need for special attention to male patients and those with comorbidities.

## Introduction

Acute coronary syndrome is characterized by the presence of atherosclerosis in the coronary arteries. This hypoperfusion leads to impaired function of portions of the heart, resulting in conditions such as ST-segment elevation myocardial infarction, non-ST-segment elevation myocardial infarction, and unstable angina [1].

Heart disease and other cardiovascular illnesses rank among the leading causes of death, accounting for approximately 18 million fatalities worldwide, or one-third of all deaths. In 2022, the global prevalence of coronary artery disease, reported from 204 countries, was 315 million cases [2]. According to data collected globally in 2019, Pakistan recorded 918 new cases and 358 deaths per 100,000 people annually, compared to global rates of 684 new cases and 240 deaths. This indicates that Pakistan faces a greater burden of cardiovascular disease than the world average [3,4]. Acute coronary syndrome must be managed as a medical emergency, requiring prompt and decisive action. Its primary classification is based on electrocardiography conducted at admission and the results of cardiac enzyme values, including troponin and creatine kinase-myocardial band. Based on electrocardiography results and troponin T and I levels, acute coronary syndrome can be categorized as either ST-segment elevation acute coronary syndrome or non-ST-segment elevation acute coronary syndrome [5,6].

ST-segment elevation acute coronary syndrome is characterized by acute chest discomfort and electrocardiogram findings of ST-segment elevation. In contrast, non-ST-segment elevation acute coronary syndrome presents with acute chest pain but lacks ST-segment elevation on the electrocardiogram. The electrocardiogram patterns in non-ST-segment elevation acute coronary syndrome may include transient or persistent ST-segment depression, T-wave inversion, T-wave flattening, or, occasionally, no discernible pattern. Non-ST-segment elevation acute coronary syndrome is further divided into two subtypes: unstable angina, where troponin levels remain within normal limits, and non-ST-segment elevation myocardial infarction, characterized by elevated troponin levels [6,7].

As discussed, a significant proportion of non-ST-segment elevation acute coronary syndrome patients may exhibit unremarkable electrocardiogram findings during their initial evaluation in the emergency department, indicating that the electrocardiogram can be inconclusive in many acute coronary syndrome cases. While individuals presenting with chest discomfort and normal electrocardiogram readings generally have a favorable prognosis regarding cardiac-related morbidity and mortality, the possibility of non-ST-segment elevation myocardial infarction should still be considered in the differential diagnosis [8]. Therefore, each patient presenting with acute coronary syndrome and a normal electrocardiogram should undergo thorough evaluation, including assessment of troponin T and I levels, to rule out myocardial damage caused by the ischemic event. This is crucial for identifying and managing potential cardiac complications. Determining the prevalence of non-ST-segment elevation myocardial infarction in acute coronary syndrome patients with a normal electrocardiogram supports the idea that acute coronary syndrome with a normal electrocardiogram can still involve myocardial damage [9-11]. While various studies report the incidence of non-ST-segment elevation myocardial infarction in different regions, data on its prevalence in Pakistan is limited. This study was designed to determine the prevalence of non-ST-segment elevation myocardial infarction in acute coronary syndrome patients with unremarkable electrocardiograms at the cardiac emergency department of Lady Reading Hospital, Peshawar. The results of this study have helped healthcare providers implement appropriate measures for diagnosing non-ST-segment elevation myocardial infarction in emergency settings.

## Materials and methods

Study design, duration, and setting 

This cross-sectional study was conducted at the cardiac emergency department of Lady Reading Hospital, Peshawar, over six months, from October 24, 2022, to April 24, 2023. The study aimed to assess the incidence of non-ST-segment elevation myocardial infarction among patients presenting with acute coronary syndrome and normal electrocardiograms in this setting.

Sample size and inclusion criteria

The sample size for this study was calculated based on the following assumptions: a precision level of 5.00%, a prevalence rate of 8.40% [12], and an infinite population size. Based on these parameters, the estimated sample size was 119. However, a total of 120 patients aged 18 years or older, who presented to the cardiac emergency department within 24 hours of acute coronary syndrome symptoms, were ultimately included in the study.

Exclusion criteria 

Patients with pre-established diagnoses of valvular heart disorders, myocardial pathologies, or congenital cardiac anomalies were excluded from the study to prevent confounding the results.

Data collection

Baseline information, including demographics and medical history, was gathered from all participants. This included details such as age, gender, body mass index, smoking history, presence of diabetes, hypertension, and duration of symptoms. An electrocardiogram was performed to detect any ST-segment elevation, and cardiac biomarkers, particularly cardiac troponin T and I levels, were assessed. Patients with troponin T levels exceeding 0.5 ng/mL were classified as having non-ST-segment elevation myocardial infarction according to the Fourth Universal Definition of Myocardial Infarction [13].

Data analysis

Statistical analysis was performed using the Statistical Package for the Social Sciences (IBM SPSS Statistics for Windows, Version 23, Armonk, NY). Categorical variables, such as gender and non-ST-segment elevation myocardial infarction status, were presented as frequencies and percentages. Continuous variables, including age, body mass index, and duration of symptoms, were reported as means and standard deviations. The effects of potential modifiers, such as age, gender, body mass index, and the presence of comorbidities, were assessed through stratification. The chi-square test was applied to assess the statistical significance of differences, with a p-value of less than or equal to 0.05 considered statistically significant. Grammar was checked using the Grammarly software (Grammarly, Inc., San Francisco, CA).

Ethics

Ethical approval for the study was obtained from the Institutional Review Board of Lady Reading Hospital under approval number 330/LRH/MTI. Patients were enrolled in the study after providing written informed consent, ensuring that all ethical guidelines were followed throughout the research process.

## Results

The results showed that the average age was 53.96 years, with a variation of 8.57 years. The average body mass index was 27.05 kg/m², with a variation of 2.59 kg/m². Patients experienced symptoms for an average of 12.03 hours before seeking treatment, with a variation of 5.07 hours. The details of patient demographics (age, body mass index, and symptom duration) are shown in Table 1.

**Table 1:** Details of patient demographics (age, body mass index, and symptom duration)

Serial no.	Patient demographics	Mean ± SD
1	Age	53.96 ± 8.57 years
2	Body mass index	27.05 ± 2.59 kg/m^2^
3	Duration of symptoms	12.03 ± 5.07 hours

The male patients were 65.83% (n = 79) of the total population, while female patients were 34.17% (n = 41). The analysis of risk factors and comorbidities among the patient group showed that 32.5% of the patients (n = 39) were smokers, and 20% (n = 24) had a family history of medical conditions. When examining comorbidities, diabetes was present in 55.83% of the patients (n = 67), hypertension in 47.50% (n = 57), and dyslipidemia in 23.33% (n = 28). The overview of risk factors, comorbidities, and gender distribution is shown in Table 2.

**Table 2:** Overview of risk factors, comorbidities, and gender distribution

Serial no.	Main category	Sub-category	No. of patients (n)	Percentage (%)
1	Gender	Male	79	65.83%
		Female	41	34.17%
2	Risk factors	Smoking	39	32.5%
		Family history	24	20%
3	Comorbidities	Diabetes	67	55.83%
		Hypertension	57	47.50%
		Dyslipidemia	28	23.33%

Of the total patient population, 32 individuals, representing 26.66%, were diagnosed with non-ST-segment elevation myocardial infarction, while 88 individuals, accounting for 73.33%, were not diagnosed with non-ST-segment elevation myocardial infarction. These values provide a clear indication of the proportion of patients affected by the condition compared to those who were not. Stratification of the prevalence of non-ST-segment elevation myocardial infarction with age, gender, body mass index, and presence of comorbidities showed a significant correlation between non-ST-segment elevation myocardial infarction and the presence of comorbidities and non-ST-segment elevation myocardial infarction and male gender as shown in Table 3. Male patients exhibited a considerably higher prevalence of non-ST-segment elevation myocardial infarction (33%) compared to female patients (14.6%), with a statistically significant p-value of 0.032, indicating gender-specific vulnerabilities. Additionally, the presence of comorbidities, such as diabetes, hypertension, and dyslipidemia, was strongly associated with the occurrence of non-ST-segment elevation myocardial infarction. Individuals with these conditions had a rate of 33%, compared to 16% in those without comorbidities, supported by a p-value of 0.043. However, neither age nor body mass index showed a significant statistical correlation with the occurrence of non-ST-segment elevation myocardial infarction in this sample.

**Table 3:** Stratification regarding demographics and comorbidities

Serial no.	Demographics and comorbidities	Subcategory	No. of patients (n)	Diagnosed with non-ST-segment elevation myocardial infarction “Yes”		Diagnosed with non-ST-segment elevation myocardial infarction “No"		p-value
				n	%	n	%	
1	Age	≤45 years	15	4	26.67%	11	73.33%	1.00
		>46 years	105	28	26.67%	77	73.33%	
2	Gender	Male	79	28	33%	53	67%	0.032
		Female	41	6	14.6%	35	85.4%	
3	Body mass index	≤25	68	15	22%	53	78%	0.19
		>25	52	17	32.7%	35	67.3%	
4	Presence of comorbidities (diabetes, hypertension, dyslipidemia)	Yes	76	25	33%	51	67%	0.043

## Discussion

The prevalence of non-ST-segment elevation myocardial infarction has been examined in multiple studies with different epidemiological risk factors and clinical histories. In a study by Tousek et al., the incidence of non-ST-segment elevation myocardial infarction in acute coronary syndrome patients was reported to be 8.4%. The researchers attributed the equal prevalence of non-ST-segment elevation myocardial infarction and ST-segment elevation myocardial infarction in this study to the large proportion of patients from high-risk groups [12].

In Pakistan, Khan et al. conducted a study assessing the burden of non-ST-segment elevation myocardial infarction in acute coronary syndrome patients with unremarkable electrocardiogram findings. This study emphasized that non-ST-segment elevation myocardial infarction should not be ruled out in patients presenting with acute retrosternal chest pain without evaluating cardiac enzyme levels. Among 215 acute coronary syndrome patients with normal electrocardiograms within 24 hours of symptom onset, the mean age was 54.3 years, and non-ST-segment elevation myocardial infarction was diagnosed in 22.8% of cases. The study highlighted the high prevalence of non-ST-segment elevation myocardial infarction with normal electrocardiogram findings, particularly in older male patients [14].

Iltaf conducted a cross-sectional study to assess the prevalence of non-ST-segment elevation myocardial infarction in acute coronary syndrome patients with normal electrocardiograms. Of the 150 participants (64.67% male, 35.33% female), the prevalence of non-ST-segment elevation myocardial infarction was 24.67% (n = 37). The study concluded a high prevalence of non-ST-segment elevation myocardial infarction in acute coronary syndrome patients with normal electrocardiograms, underscoring the global significance of coronary artery disease as a major cause of morbidity and mortality [15].

Ahmad et al. examined the frequency of non-ST-segment elevation myocardial infarction in patients with atypical electrocardiogram findings. This study included 257 patients (60.3% male, 39.7% female) with a mean age of 56.33 ± 8.07 years and an average body mass index of 27.28 ± 5.25 kg/m². Comorbidities included hypertension (60.3%) and diabetes mellitus (42.4%), with non-ST-segment elevation myocardial infarction diagnosed in 30% of the population [16].

A systematic review by Khan et al. highlighted the increasing prevalence of coronary artery disease and myocardial infarction, emphasizing that acute myocardial infarction mortality correlates with treatment delays and misdiagnoses. Atypical myocardial infarction presentations, which are difficult to diagnose, significantly impact morbidity and mortality. These cases often involve patients over 50 with multiple comorbidities (diabetes, hypertension, dyslipidemia, substance abuse) presenting with diverse symptoms such as abdominal, head, or neck pain, shortness of breath, dizziness, fatigue, syncope, or gastrointestinal discomfort. Emergency and primary care physicians should maintain a high index of suspicion for atypical myocardial infarction in such patients [17].

In our study, the mean age of patients was 53.96 ± 8.57 years. The male gender accounted for 65.83% of the total study population, while females were 34.17%. The incidence of non-ST-segment elevation myocardial infarction was present in 32 (26.66%) of the patients. Stratification of non-ST-segment elevation myocardial infarction incidence did not establish any correlation with age or body mass index; however, a significant correlation was found with male gender (p = 0.032) and comorbidities, including diabetes mellitus, hypertension, and dyslipidemia (p = 0.043). These findings align with the studies discussed earlier, emphasizing the importance of diagnosing non-ST-segment elevation myocardial infarction with cardiac biomarkers, even in patients with normal electrocardiograms [12-17].

One of the limitations of this research is the small sample size. Additionally, the study only included patients from a single cardiac emergency center. Future research with larger, multicenter studies will contribute valuable data on the prevalence of non-ST-segment elevation myocardial infarction.

## Conclusions

In conclusion, a considerable number of patients who presented with symptoms similar to acute coronary syndrome but had normal electrocardiograms were later diagnosed with non-ST-segment elevation myocardial infarction. This underscores the importance of assessing cardiac biomarkers in all suspected cases of acute coronary syndrome, even when initial electrocardiogram results appear normal. Healthcare professionals should remain especially alert to the possibility of non-ST-segment elevation myocardial infarction, particularly in male patients and those with underlying conditions such as diabetes, hypertension, and high cholesterol.
